# Reinforcement-Sensitive Personality Traits Associated With Passion in Heterosexual Intimate Relationships: An fNIRS Investigation

**DOI:** 10.3389/fnbeh.2020.00126

**Published:** 2020-07-21

**Authors:** Li Gu, Ruoxi Yang, Qihan Zhang, Peng Zhang, Xuejun Bai

**Affiliations:** ^1^Department of Psychology, School of Humanities and Management, Guangdong Medical University, Dongguan, China; ^2^Faculty of Psychology, Tianjin Normal University, Tianjin, China; ^3^Center of Collaborative Innovation for Assessment and Promotion of Mental Health, Tianjin, China

**Keywords:** reinforcement-sensitive personality traits, passion, functional near-infrared spectroscopy (fNIRS), reward sensitivity, ventrolateral prefrontal cortex

## Abstract

According to the triangular theory of love, passion is an indispensable component of romantic love. Some brain imaging studies have shown that passionate arousal in intimate relationships is associated with the reward circuits in the brain. We hypothesized that the individual reward sensitivity trait is also related to passion in intimate relationships, and two separate studies were conducted in the present research. In the first study, 558 college students who were currently in love were selected as participants. The correlation between intimacy and reinforcement sensitivity in individuals identifying as heterosexual was explored using the Sensitivity to Punishment and Sensitivity to Reward Questionnaire, the Passionate Love Scale, and the Triangular Love Scale. In the second study, participants were 42 college students who were also currently in love. Functional near-infrared spectroscopy (fNIRS) was adopted to explore the neurophysiological interaction between reward sensitivity and emotional arousal induced in participants when presented a photograph of their partner, a friend, or a stranger. The results showed that reward sensitivity was positively correlated with passion, and punishment sensitivity was negatively correlated with intimacy and commitment. Significant interactions between reward sensitivity and photograph type were found, and the triangular part of the inferior frontal gyrus showed a particular relevance to the reward-sensitive personality trait toward partners. Overall, the findings support reinforcement sensitivity theory and suggest that reinforcement-sensitive personality traits (personality traits of reward and punishment sensitivity) are associated with all three components of love, with only reward sensitivity being related to passion.

## Introduction

According to the triangular theory of love, there are three components of love: passion, intimacy, and commitment (Sternberg, 1986). All three components are liable to change over time, among which passion is the most variable (Ahmetoglu et al., 2010). Passion refers to a strong desire toward another person, including sexual arousal (Sternberg, 2006). Neurophysiological studies have shown that the element of sexual desire in passion is both independent and measurable (Diamond and Dickenson, 2012). Two fMRI studies have shown that the brain regions activated by passion differ not only from the regions activated by admiration and commitment but also from those activated by sexual arousal (Aron et al., 2005; Xu et al., 2011). The concept of passion without a sexual element is aligned with the definition of Vallerand, in which passion is considered to be an intense propensity toward our beloved that makes us think of them as highly important and therefore makes us willing to invest our time and energy in our relationship with them (Vallerand et al., 2003). Highly passionate individuals tend to evaluate their lovers as more attractive and trustworthy (Gunaydin and DeLong, 2015). However, few studies have examined what kinds of people show higher passion in intimate relationships. The objective of our research is to focus on passion without the sexual element by using an experimental paradigm of passion based on previous studies that effectively controls for sexual arousal and to explore the relationship between personality traits and passion in intimate relationships.

Based on Eysenck’s theory of personality, proposed reinforcement sensitivity theory (RST) and later published a revised version of his theory (Gray and Mcnaughton, 2000). According to the revised theory, there are three interactive, neurobiologically valid systems that influence behavior. The behavioral activation system (BAS) is activated by conditioned and unconditioned appetitive signals of reward or non-punishment, and it initiates approach behavior and results in positive emotional experiences. The fight, flight, and freeze system (FFFS) is activated by conditioned and unconditioned aversive stimuli, and it is associated with feelings of rage and fear. The behavioral inhibition system (BIS) is activated by conflict between the BAS and FFFS, and it is associated with feelings of anxiety (McNaughton and Corr, 2004; Smillie et al., 2006). From the perspective of personality traits, punishment sensitivity reflects the combined function of the FFFS and BIS, and reward sensitivity reflects BAS functioning (Corr, 2004). Several fMRI studies in recent years have provided support for the neurophysiological basis of the RST by linking punishment and reward sensitivity to anatomical differences in the limbic and frontostriatal regions (Adrián-Ventura et al., 2019a, b). Reward- and punishment-sensitive personality traits can be also measured by self-report measures consisting of punishment and reward sensitivity scales developed based on the RST (Carver and White, 1994; Torrubia et al., 2001). At present, the RST is widely used in research on addiction behaviors and human resource management, as well as predictive studies of psychological disorders and mental diseases (Hutchison et al., 2013; Schreurs et al., 2014; Hemel-Ruiter et al., 2015) but it has received little attention in research on intimate relationships.

Individuals with passion in intimate relationships are strongly attracted to their partners; they are willing to approach them and invest the time and energy for their partners. This tendency to approach partners appears similar to the approach behavior of reward sensitive individuals in the RST theory. Unlike intimacy and commitment, passion in intimate relationships reflects excitatory emotional arousal, in which individuals attribute the cause of their emotional arousal to their partner (Berscheid and Walster, 1974). In the classic suspension bridge experiments, male passersby were approached on either a fear-arousing suspension bridge or a non-fear-arousing bridge by an attractive female interviewer who asked them to fill out questionnaires containing Thematic Apperception Test (TAT) pictures. The results showed that the sexual content of stories written by subjects on the fear-arousing bridge and the tendency of these subjects to attempt post-experimental contact with the female interviewer were both significantly greater, indicating that emotional arousal in dangerous situations tends to increase people’s physiological response, which they can mistake or misattribute as passion toward strangers, leading to increased approaching behavior (Dutton and Aron, 1974). Although the “suspension bridge effect” can explain the increasing approaching behavior well, in addition, we think that the people who are more likely to cross dangerous suspension bridges may also be risk-taking individuals, who have higher passionate arousal levels and higher levels of approaching behavior toward others. Previous studies have shown that reward-sensitive traits are related to risk-taking behaviors (Romer et al., 2016) and overactive reward circuits increase adolescents’ risk-taking behavior (Galvan et al., 2006; Van Leijenhorst et al., 2010). According to RST, individuals with a reward-sensitive personality are more likely to engage in approach behavior, which is very similar to the increase in passionate approach behavior under emotional arousal. However, individuals with a punishment-sensitive personality are more likely to engage in inhibition behavior and risk aversion, which is unlikely to contribute to increased passionate arousal. On this basis, we propose the hypothesis that passion is related to an individual’s reward sensitivity traits—individuals with high reward sensitivity are more likely to have a higher level of passion in intimate relationships.

Some brain imaging studies have confirmed a correlation between the reward circuits in the brain and passionate arousal in intimate relationships. For example, Aron et al. (2008) found that passionate arousal is regulated by the neurotransmitters dopamine and serotonin, which control reward emotion in specific brain regions. Activation of the dopamine-rich reward center in partners who had been married more than 10 years and reported still being in love with each other was observed when participants were presented a photograph of their partner (Acevedo et al., 2012). These results are consistent with previous studies of early stage romantic love being associated with activation in the right ventral tegmental area (VTA) and caudate, as was observed when the partners first fell in love and consistent with their subjective reports (Bartels and Zeki, 2000; Aron et al., 2005; Ortigue et al., 2007; Xu et al., 2011). Two fMRI studies have shown that passion-related brain regions overlap with the reward-motivated brain regions, such as the right medial caudate nucleus and other dopamine-focused reward-stimulating brain regions (Bartels and Zeki, 2004; Aron et al., 2005). Research by Fisher et al. (2016) also using fMRI, supports this view: Feelings of intense romantic love engage regions of the brain’s reward system, most specifically the dopamine-rich regions, including the ventral tegmental area, which is also activated during drug and/or behavioral addiction. This is because the experience of romantic love shares reward pathways with a range of substance and behavioral addictions. Addiction behavior is closely related to the BAS, which is activated by reward signals and triggers approach behavior (Hemel-Ruiter et al., 2015). In summary, these studies have confirmed that passionate arousal in intimate relationships is closely related to reward mechanisms at the neurophysiological level.

Functional near-infrared spectroscopy (fNIRS) is a recently developed functional brain imaging technique that has been used in a wide variety of fields, including behavioral studies, neuroscience, sports medicine, and brain–computer interfaces (BCI) (Bhutta et al., 2015; Naseer and Hong, 2015; Hong et al., 2017; Zhang et al., 2019). fNIRS is particularly suitable for personality studies because it can investigate cortical responses in a natural setting, compared with other functional brain imaging techniques like PET and functional MRI (Boas et al., 2004; Sato et al., 2007). Due to its fine time resolution, subjects under examination can speak in a sitting position, with their eyes open. fNIRS addresses one of the main problems of functional neuroimaging personality studies: the lack of a natural setting during neuroimaging (Sato et al., 2012).

Based on the previous research, we speculated that individuals with different reward sensitivities would differ in their subjective reports of passion levels in intimate relationships, and these differences would be reflected in prefrontal cortex activation patterns. In the first study, we verified the correlation between passion level and reinforcement sensitivity. In the second study, we adopted fNIRS to explore the neurophysiological interactions between reward sensitivity and emotional arousal.

## Study 1

### Materials and Methods

#### Participants

We recruited 812 participants in this questionnaire survey, and the recruitment requirements stated that the participants should be college students who were currently in love. In total, 256 participants completed the questionnaires through a scene questionnaire. The rest of the participants were recruited by advertising on the university campuses in several cities. A total of 556 college students contacted us expressing interest in this questionnaire survey. We then sent out the questionnaires, certificate of ethics, and informed consent form via email. In total, 676 valid questionnaires were returned, and 126 invalid questionnaires were excluded because the duration of the current relationship exceeded more than 3 years. Thus, 550 valid questionnaires were obtained for analysis, reflecting an effective rate of 81.36%. There were 254 male and 296 female participants, with mean ages of 19.45 ± 1.27 and 20.07 ± 1.71 years, respectively, and the mean duration of the relationship was 12.45 ± 10.42 months. This study was approved by the Ethics Committee of Tianjin Normal University. Informed consent was obtained from all participants.

#### Questionnaires

##### Sensitivity to punishment and sensitivity to reward questionnaire

The SPSRQ is a commonly used self-report questionnaire of reinforcement sensitivity. The scale was compiled by Torrubia et al. (2001) and has a total of 48 yes/no items. It consists of two subscales of 24 items each: sensitivity to punishment (hereinafter referred to as SP) and sensitivity to reward (hereinafter referred to as SR). Even items belong to SR and odd items to SP. Scores for each scale can be obtained by adding up all the “yes” answers. The coefficients of internal consistency for the two subscales were found to be 0.83 and 0.76, with the three-month retest reliability being 0.81 and 0.87 for the SP and SR, respectively, (Torrubia et al., 2001). In this study, the coefficients of internal consistency for the SP and SR subscales were 0.84 and 0.77, respectively.

##### Triangular love scale

The TLS is a 45-item scale developed by Sternberg (1997) consisting of three subscales of intimacy, passion, and commitment. The first 15 items in the scale reflect intimacy, the second 15 measure passion, and the final 15 reflect commitment. A seven-point Likert-type scale is used to rate the degree of agreement (from 1, “Not at all,” to 7, “Extremely”). The scores for each group of 15 items are added up to obtain the total score. High scores represent a higher level of intimacy, passion, and commitment. The coefficients of internal consistency of the three subscales were found to be 0.91, 0.94, and 0.94, respectively, (Sternberg, 1997). The internal consistency coefficients for the intimacy (hereinafter referred to as TLS-I), passion (hereinafter referred to as TLS-P), and commitment (hereinafter referred to as TLS-C) subscales in this study were 0.94, 0.90, and 0.94, respectively.

##### Passionate love scale

The PLS was compiled by Hatfield and Sprecher (1986) to measure the passion level of individuals in romantic relationships. The scale contains a total of 30 items, using a nine-point scoring method, with a higher overall score representing a higher level of passion. The internal consistency of the scale found by the scale developers was 0.94. The coefficient of internal consistency was 0.94 in this study.

#### Data Analysis

SPSS 22.0 was used to conduct the descriptive statistics and correlation analyses on the demographic variables and scores obtained from the SPSRQ, TLS, and PLS.

### Results

#### Descriptive Statistics and Correlation Analyses

Table 1 shows the descriptive statistics for the demographic and major variables, as well as the correlation analyses between the variables. Sensitivity to reward was positively correlated with both PLS (*r* = 0.129, *p* < 0.01) and TLS-P (*r* = 0.139, *p* < 0.01)—that is, individuals with higher sensitivity to reward had higher passion levels. A positive correlation was also found between reward sensitivity and the number of previous relationships (love experiences) (*r* = 0.098, *p* < 0.05), with a higher reward sensitivity corresponding to a higher number of previous relationships. However, sensitivity to punishment was negatively correlated with TLS-I (*r* = −0.167, *p* < 0.01) and TLS-C (*r* = −0.142, *p* < 0.01), indicated individuals with higher punishment sensitivity had lower self-reported levels of intimacy and commitment toward their partners.

**TABLE 1:** The mean values, standard deviations and correlation coefficients of the demographic and questionnaire variables.

Variables	*M*	*SD*	Items	1	2	3	4	5	6	7	8
1. Experiences	2.72	1.85	–	1							
2. Duration	12.45	10.42	–	–0.058	1						
3. SR	11.29	4.09	24	0.098*	–0.03	1					
4. SP	12.00	5.26	24	–0.023	0.049	0.243**	1				
5. TLS-I	90.03	11.91	15	0.136**	0.135**	–0.05	−0.167**	1			
6. TLS-P	77.35	14.31	15	0.08	0.106*	0.139**	–0.044	0.622**	1		
7. TLS-C	89.76	12.56	15	0.057	0.126**	–0.083	−0.142**	0.740**	0.713**	1	
8. PLS	203.66	28.56	30	0.088*	0.094*	0.129**	–0.027	0.616**	0.965**	0.694**	1

*p < 0.05 **p < 0.01. Experiences: The number of previous intimate relationships.

### Discussion

#### Reward Sensitivity and Passion

The results obtained from the two measures of passion (the PLS and TLS) showed that only reward sensitivity was positively correlated with passion, while punishment sensitivity was negatively correlated with intimacy and commitment. These results confirm our hypothesis that passion is only related to the reward-sensitive personality trait and not the punishment-sensitive personality trait. From a physiological perspective, passion is a driving force that is completely different from intimacy and commitment. According to the classic analysis of love, passion arises from two factors: emotional arousal and the belief that the loved one is the cause of the emotional arousal (Berscheid and Walster, 1974). Combined with Gray’s revised theory (Gray and Mcnaughton, 2000) the BAS mediates reactions to appetitive stimuli (conditioned and unconditioned appetitive signals of reward) and initiates approach behavior. Individuals with high reward sensitivity (strong BAS) should be most reactive to reward, which would result in more approach behavior relative to individuals with low reward sensitivity (weak BAS). Highly passionate individuals are more likely to engage in approach behavior under emotional arousal, as in the “suspension bridge effect” (Dutton and Aron, 1974) which is very similar to high reward sensitivity.

#### Punishment Sensitivity and Intimacy/Commitment

In this study, punishment sensitivity was correlated with intimacy and commitment but not passion. Intimacy includes common features in love relationships such as understanding, communication, support, and sharing. Commitment refers to the determination to commit to love and to strive to maintain it (Sternberg, 1986). Compared with passion, which is a driving force, intimacy and commitment seem to be more susceptible to the influence of interactive interpersonal relationships. According to the revised theory, the FFFS is responsible for mediating reactions to all aversive stimuli, initiates avoidance and escape behavior, and is associated with the emotion of fear. The BIS is responsible for resolving goal conflict (e.g., between the BAS [approach] and FFFS [avoidance]), and this process generates the state of anxiety (Gray and Mcnaughton, 2000). Punishment sensitivity reflects combined FFFS/BIS functioning, but the FFFS is more associated with general punishment sensitivity (Corr, 2004). Individuals with high punishment sensitivity (strong FFFS/BIS) should be most reactive to punishment and exhibit more avoidance behavior and risk aversion relative to individuals with low punishment sensitivity (weak FFFS/BIS). Hence, punishment sensitivity is unlikely to contribute to increased passionate arousal. The FFFS is particularly associated with rage, fear, and behavioral initiation (e.g., partner shows active avoidance of an aversive stimulus or starts a fight when confronted with an aversive stimulus in an intimate relationship). Previous studies have shown that individuals with high levels of avoidance behavior often suspect others and rarely share their feelings, thoughts, and hopes with their partner (Ein-Dor et al., 2011). When asked for comfort and support by a partner, such individuals act more negatively and sometimes even become annoyed, reducing their intimacy (Campbell et al., 2001) and commitment (Mikulincer and Shaver, 2007) to their partner, resulting in a decline in the quality of their intimate relationships.

In summary, the results of study 1 support the RST, and the personality traits of reward and punishment sensitivity are associated with all three components of love. Unlike intimacy and commitment, passion is uniquely related to reward sensitivity.

## Study 2

### Materials and Methods

#### Participants

Participants in Study 2 included 46 college students who were currently in love, and the duration of the current relationship was no more than 3 years. We recruited the students by posting recruitment advertisements on university campuses. The sample was separate from that recruited in Study 1. Four participants were not included in the analyses due to large motion artifacts, and therefore, the final analysis dataset comprised 42 participants, with a mean age of 21.96 ± 2.42 years. According to the scores for the SR subscale of the SPSRQ, the participants were divided into two groups: the high reward sensitivity group (SR-H, *n* = 21, 12 male and 9 female) and low reward sensitivity group (SR-L, *n* = 21, 8 male and 13 female), with mean SR subscale scores of 13.14 ± 1.82 and 7.95 ± 2.16, respectively, and *t*-test results confirmed that there was a significant difference between the two groups (*t* = 8.422, *df* = 40, *p* < 0.001). The participants were all right-handed, with normal vision or corrected visual acuity and no history of mental illness. Additionally, no reported conflict with their partners during the week before the visit was noted. This study was approved by the Ethics Committee of Tianjin Normal University. Each participant provided written informed consent prior to starting the experiment and obtained a cash reward after the experiment.

#### Experimental Equipment

An fNIRS system manufactured by Shimadzu Corporation (LABNIRS/16, Shimadzu Corporation, Kyoto, Japan) was used. The brain activity in the cerebral cortex was monitored by three semiconductor lasers with wavelengths of 780 ± 5 nm, 805 ± 5 nm, and 830 ± 5 nm, respectively, and then transformed into changes in the cortical hemoglobin concentration using the modified Beer–Lambert law (MBLL). The hemoglobin concentrations were measured based on three indicators: oxyhemoglobin (HbO), deoxyhemoglobin (HbR), and total hemoglobin (HbT). HbO has been found to be more sensitive to changes in brain activity during task simulation than HbR and HbT (Hoshi et al., 2001). Therefore, change in cerebral cortex HbO concentration were selected in this study as an analysis indicator (with a sampling rate of 11 Hz).

#### Experimental Materials

We took experimental photographs of the participants’ partners and friends. The experimental photographs were taken at a distance of 1 m of individuals with neutral expressions, unexposed teeth, and no makeup or accessories. Three of the partners and two of the friends were not in the local area, so they were asked to provide the photos taken as required. Adobe Photoshop was used to process all the photographs, controlling the brightness and contrast, and removing facial marks such as nevi and spots. The experimental material was presented on a 17-inch display with a resolution of 2048 × 1536 pixels. All the pictures were digitized and presented in black and white with the same brightness and contrast; they were also all the same size. All photographs were frontal pictures taken above the neck only.

In order to avoid participants being influenced by the appearance of strangers, including factors such as beauty or eccentricity, the final experimental materials of two photographs of strangers (one male and one female) were selected in advance by 30 graders (15 male and 15 female) from 16 students (8 male and 8 female) who volunteered to provide photographs as experimental materials. They were asked to evaluate the photographs of each stranger’s appearance from high to low and select the most “average” or generic photos of strangers. The Kendall coefficient for the photograph evaluation (*W* = 0.87) showed good consistency.

#### Experimental Design

A mixed 2 (type of participant) × 3 (photo type) design was used. Type of participant was the between-subjects variable, consisting of high and low reward sensitivity. Photo type was the within-subjects variable, consisting of three levels: partner, friend, and stranger. It has been shown that greater intensity of passionate love felt for one’s partner is associated with greater accuracy in correctly identifying the partner’s face from those of friends and strangers (Nakamura et al., 2017). Individuals scoring high in passion also tend to evaluate their lover’s photos as more attractive and trustworthy (Gunaydin and DeLong, 2015). Therefore, we used the same experimental paradigm to evoke passion through the recall of events related to a partner’s photograph that was commonly used by brain imaging researchers in the field of romantic love (Aron et al., 2005; Xu et al., 2011).

#### Experimental Procedures

**FIGURE 1:**
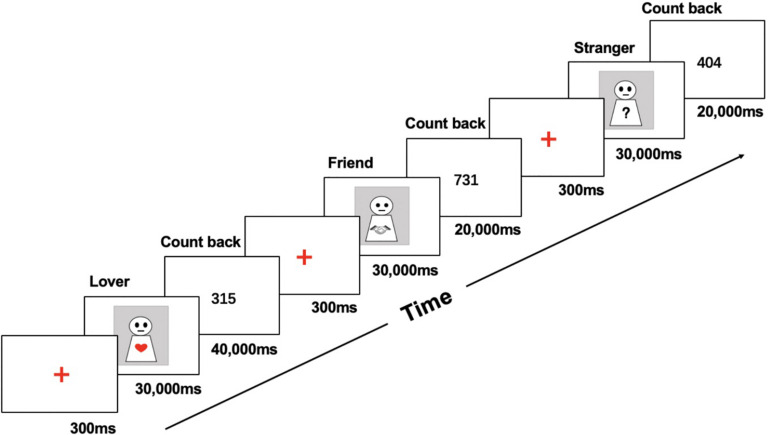
Experimental procedure.

After arrival, the participants took a 5-min rest in the laboratory, and then the experimental instruments were placed on the participant after they had adapted to the room temperature. Before the formal start of the experimental procedure, participants were allowed to sit in silence for 3 min, and baseline values of HbO concentration data were collected. After 3 min of empty screen presentation, the screen showed the first focus, with the experimental procedure completed as described below (see Figure 1).

(1) Stimuli presentation: After presenting a 300-ms focus point in the middle of the screen, participants were presented with photographs of their partners, friends, and strangers. The participants were required to recall their happy shared experiences when photographs of partners and friends were shown and were asked to avoid sexual aspects when recalling partner-related events (Aron et al., 2005; Xu et al., 2011). When presented pictures of strangers, they were asked to avoid recalling friend- or partner-related events. The presentation time for each photo was 30,000 ms.
(2) Distraction task: A random number was presented in the gap between the stimuli, and the participants were asked to complete a backward counting task. This task required participants to continue subtracting 7 from the number presented (e.g., 842-7 = 835-7 = 828-7 = …) until the number disappeared. The presentation duration of the number was 20,000 ms after the photographs of friends/strangers and 40,000 ms after the photographs of partners. This difference in the duration of the distraction task was implemented because it takes longer to eliminate emotional responses to photographs of partners (highly arousing stimuli) compared to photographs of friends and strangers (low-arousing stimuli), thus restoring the neurophysiological baseline and avoiding a spillover effect (Aron et al., 2005). The starting numbers of each distraction task stage were randomly generated.

The above six blocks were repeated six times, and the experimental procedure lasted approximately 17 min. The order of the first-stage stimulus pictures (low or high arousal) was balanced among participants. After the experiment, the participants completed a simple recorded interview in which they were asked to describe the events that they had recalled as well as the general content of these events, and whether they had performed the counting backward task during the distraction task stage.

#### Probe Arrangement

**FIGURE 2:**
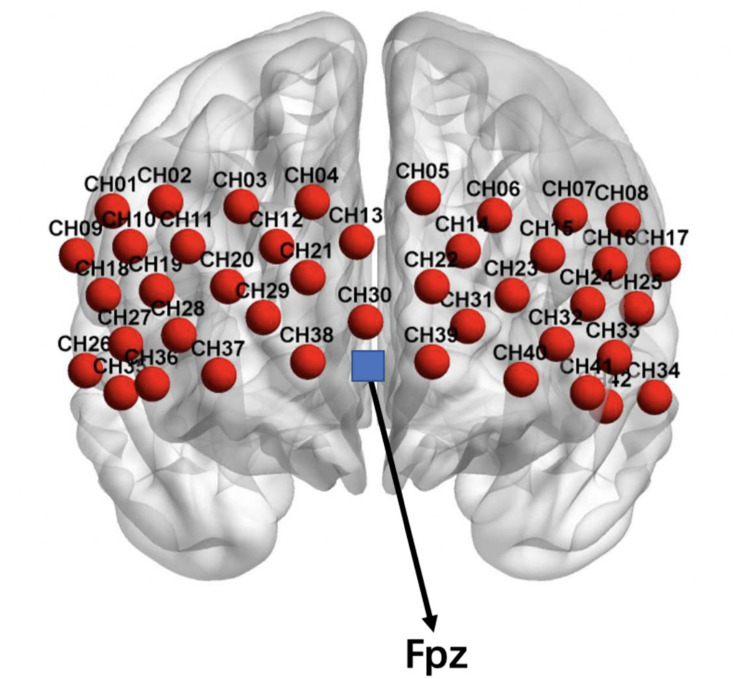
Layout of the fNIRS channels. The blue block represents the Fpz position.

A multi-channel layout consisting of a 3 × 9 arrangement was adopted, using 14 emitters and 13 detectors. Two types of probes were distributed alternately with an adjacent spacing of 3 cm, thus forming a total of 42 channels (see Figure 2). The probes were placed according to the international 10–20 EEG system; thus, the T8 probe was located at the Fpz point and arranged at the prefrontal lobe of the brain. We chose the prefrontal cortex as the measured region because previous studies have reported that the prefrontal cortex is related to both personality and reward (Turner et al., 2003; Van Leijenhorst et al., 2010; Sato et al., 2012; Ikeda et al., 2014; Oonishi et al., 2014). Using the 3D locator, the correspondence between the Montreal Neurological Institute space coordinates and Anatomical Automatic Labeling (AAL) was obtained by probability registration (Jang et al., 2009). Refer to the corresponding positions of the maximum coverage of each channel listed in Supplementary Material for further details.

#### Data Analysis of the fNIRS Signals

The data were processed with the NIRS-Statistical Parametric Mapping (NIRS-SPM) toolbox (version; v.4.1) using the MATLAB operating environment (Ye et al., 2009). The extracted fNIRS signal was denoised and drifted by using the wavelet minimum description length method (Jang et al., 2009). Then the drift and artificial noises (such as head motion) were excluded using hemodynamic response functions (HRF). The degree of the reaction induced by the experimental tasks in response to the reference wave (beta value) on each channel was evaluated by the general linear model (GLM) to obtain the fitting coefficient β, and the temporal autocorrelation of this process was adjusted using the pre-coloring method.

According to the experimental design, SPSS 22.0 was used to perform repeated-measures analysis of variance (ANOVA) tests for statistical analysis of β under different task conditions and for different channels. All the interaction *p*-values of the 42 channels were subjected to false discovery rate (FDR) correction, which is widely adopted in functional neuroimaging in order to avoid Type I error in the simultaneous testing of a large number of channels (Singh and Dan, 2006). As such, only the significant channels (*p* < 0.05) were retained. Following ANOVA of each significant (FDR corrected) channel, Bonferroni correction was performed to control the type I error rate in the simple effect analysis and post hoc comparisons. Figures 5, 6 depict the variation of averaged HbO fitting β coefficients with their standard errors as determined by a two-way ANOVA when the high/low reward sensitivity groups looked at different types of photographs.

### Results

The variation of the HbO fitting coefficient β was analyzed by a 2 (group: SR-H and SR-L) × 3 (photo type: stranger, friend, and partner) ANOVA when the high/low reward sensitivity groups looked at different types of photographs. The following results were observed:

(1) The main effect of subject groups was not significant.
(2) The main effect of photo type was significant.

**FIGURE 3:**
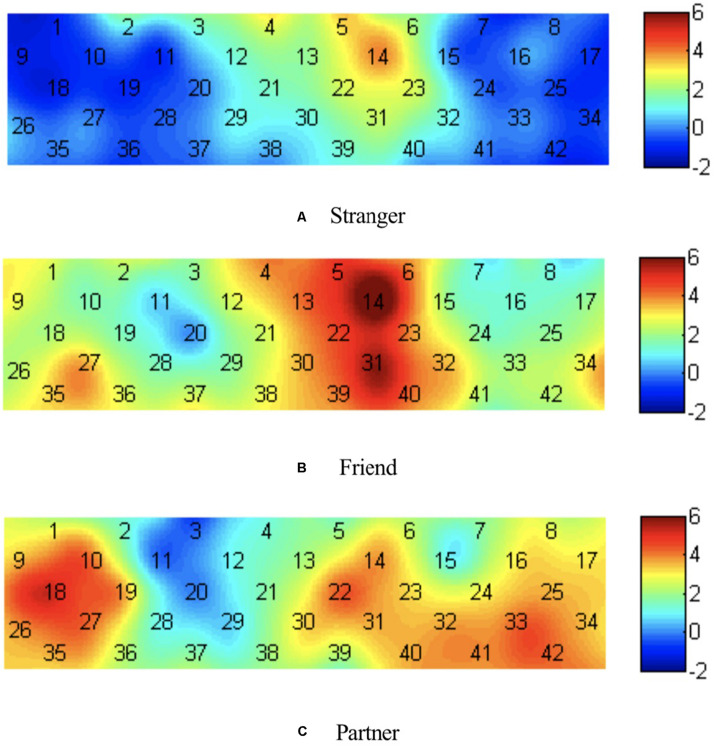
Heat map of *t* value for brain activation of different photographs. **(A)** Viewing stranger photographs; **(B)** viewing friend photographs; **(C)** viewing partner photographs.

Single-sample *t*-tests were performed for brain activation of different photographs, and the test value was 0. The channels that were activated when individuals viewed stranger photographs on channels 4, 5, and 14, corresponding to the medial orbital part of the superior frontal gyrus (Frontal_Sup_Medial) and superior frontal gyrus (Frontal_Sup). The channels activated when individuals viewed friend photographs were channels 1, 2, 4–6, 9, 12–14, 21–23, 27, and 30–40, corresponding to the precentral gyrus (Precentral), middle frontal gyrus (Frontal_Mid), Frontal_Sup_Medial, Postcentral gyrus (Postcentral), Frontal_Sup, triangular part of the inferior frontal gyrus (Frontal_Inf_Tri), superior temporal gyrus (Temporal_Sup), temporal pole of the superior temporal gyrus (Temporal_Pole_Sup), orbital part of the inferior frontal gyrus (Frontal_Inf_Orb), orbital part of the middle frontal gyrus (Frontal_Mid_Orb), and orbital part of the superior frontal gyrus (Frontal_Sup_Orb). The channels that were activated when individuals viewed partner photographs were channels 1, 8–10, 14, 16–19, 22–27, 30–36, and 40–42, corresponding to Precentral, Postcentral, Frontal_Inf_Tri, Frontal_Sup, Frontal_Mid, Temporal_Sup, Frontal_Inf_Tri, Frontal_Sup_Medial, Temporal_Pole_Sup, Frontal_Inf_Orb, and Frontal_Mid_Orb, respectively. According to the *t*-value of each channel, the heat map was drawn, as shown in Figure 3.

Bonferroni post hoc comparisons revealed greater activation of channels 9 (*p* = 0.015), 10 (*p* = 0.007), 18 (*p* = 0.001), 19 (*p* = 0.004), and 42 (*p* = 0.003), corresponding to Postcentral, Frontal_Inf_Tri, Frontal_Mid, and Temporal_Pole_Sup, when individuals were looking at partner photographs compared with stranger photographs and greater activation of channel 9 (Postcentral) (*p* = 0.017) when they were looking at friend photographs compared with stranger photographs (Table 2).

**TABLE 2:** Channels with significant main effects for photograph type.

Channel	AAL	Hemisphere	Overlap (%)	*F*	*p*	*q*_(FDR)_	*Partial Eta^2^*
CH9	Postcentral	right	72.01	6.38	0.003	0.025	0.14
CH10	Frontal_Inf_Tri	right	93.08	6.84	0.002	0.028	0.15
CH18	Frontal_Inf_Tri	right	48.08	8.28	0.001	0.042	0.17
CH19	Frontal_Mid	right	61.29	6.86	0.002	0.042	0.15
CH42	Temporal_Pole_Sup	left	50.71	6.81	0.002	0.021	0.15

**FIGURE 4:**
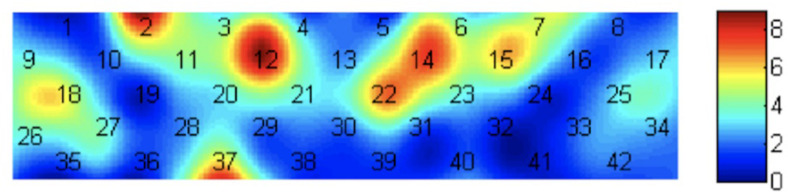
Heat map of the *F* value significant interaction between group and photograph type.

(3) Significant interactions between group and photograph type were also found (Table 3 and Figure 4).

**TABLE 3:** Channels with a significant interaction between group and photograph type.

Channel	AAL	Hemisphere	Overlap (%)	*F*	*p*	*q*_(FDR)_	*Partial Eta^2^*
CH18	Frontal_Inf_Tri	right	48.08%	5.94	0.004	0.024	0.13
CH2	Frontal_Mid	right	82.20%	9.0	0.000	0.012	0.19
CH7	Frontal_Mid	left	91.10%	5.34	0.007	0.037	0.12
CH12	Frontal_Sup	right	71.05%	8.92	0.000	0.007	0.18
CH14	Frontal_Sup	left	97.71%	7.66	0.001	0.011	0.16
CH15	Frontal_Mid	left	99.57%	6.37	0.003	0.021	0.14
CH22	Frontal_Sup	left	74.26%	7.06	0.002	0.017	0.15
CH37	Frontal_Mid_Orb	right	61.15%	8.02	0.001	0.014	0.17

**FIGURE 5:**
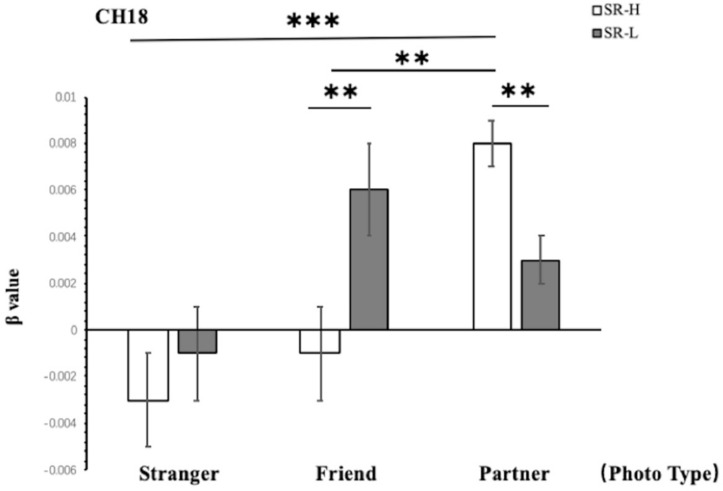
Two-way ANOVA results for CH18. SR-H for the high reward sensitivity group, SR-L for the low reward sensitivity group. Error bars represent standard errors. ^∗^*p* < 0.05, ^∗∗^*p* < 0.01, ^∗∗∗^*p* < 0.001.

According to simple effect analysis (Bonferroni) on Frontal_Inf_Tri (Channel 18), which is in the VLPFC, the activation in the high reward sensitivity group was significantly higher than that in the low reward sensitivity group when they were looking at partner photographs (*p* = 0.008), and the activation in the low reward sensitivity group was significantly higher than that in the high reward sensitivity group when they were looking at friend photographs (*p* = 0.004). Meanwhile, compared with viewing photographs of strangers (*p* < 0.001) and friends (*p* = 0.001), individuals with high reward sensitivities showed greater activation when they were looking at partner photographs. This indicates that reward sensitivity in this brain region is closely related to passion. The reactions of participants with high reward sensitivity toward partners are different not only from those to strangers but also those to friends. Excluding the relationship between familiarity and the activation of brain regions, this also verifies the significant difference in patterns of activation associated with passionate arousal in this brain region toward partners for participants with different levels of reward sensitivity (Figure 5).

**FIGURE 6:**
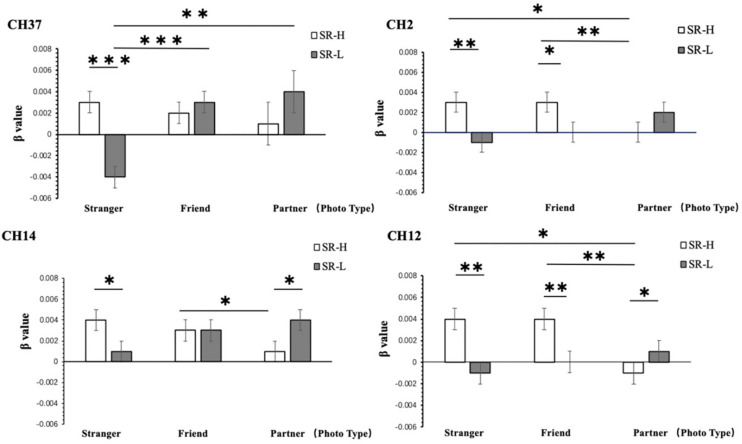
Two-way ANOVA results for CH2, CH12, CH14, and CH37. SR-H for the high reward sensitivity group, SR-L for the low reward sensitivity group. Error bars represent standard errors. ^∗^*p* < 0.05, ^∗∗^*p* < 0.01, ^∗∗∗^*p* < 0.001.

According to simple effect analysis (Bonferroni) of the other channels, the activation in the high reward sensitivity group was significantly higher than that in the low reward sensitivity group when they were looking at photographs of strangers on channels 2 (*p* = 0.001), 7 (*p* = 0.011), 12 (*p* = 0.003), 14 (*p* = 0.031), 15 (*p* = 0.005), 22 (*p* = 0.012), and 37 (*p* < 0.001), corresponding to the Frontal_Mid, Frontal_Sup, and Frontal_Mid_Orb, which are mainly in the DLPFC and the frontopolar cortex (FPC). The activation in the high reward sensitivity group was significantly lower than that in the low reward sensitivity group when they were looking at photographs of partners on channels 12 (*p* = 0.038) and 14 (*p* = 0.038), corresponding to Frontal_Sup. Meanwhile, individuals with high reward sensitivities showed greater activation when they were looking at photographs of strangers compared with partners on channels 2 (*p* = 0.028) and 12 (*p* = 0.018), corresponding to Frontal_Mid and Frontal_Sup, and also showed greater activation when they were looking at photographs of friends compared with those of partners on channels of 2 (*p* = 0.008), 12 (*p* = 0.001), 14 (*p* = 0.022), 15 (*p* = 0.038), and 22 (*p* = 0.033), corresponding to Frontal_Mid and Frontal_Sup. Individuals with low reward sensitivity showed lower activation when they were looking at photographs of strangers compared with photographs of partners on channels of 7 (*p* = 0.001), 15 (*p* = 0.022), 22 (*p* = 0.031), and 37 (*p* = 0.004), corresponding to Frontal_Mid, Frontal_Sup, and Frontal_Mid_Orb, and compared with photographs of friends on channel 37 (Frontal_Mid_Orb) (*p* < 0.001). The channels associated with the four most significant interactions are illustrated in Figure 6. The activation patterns of these channels were as follows: high and low reward sensitivity was correlated with the degree of familiarity (photographs of strangers: SR-High > SR-Low; photographs of partners: SR-Low > SR-High). In individuals with low reward sensitivity, the higher the familiarity, the lower the activation (Partners > Friends > Strangers). Conversely, in individuals with high reward sensitivity, the lower the familiarity, the higher the activation (Strangers > Friends > Partners). These channels are all distributed in the superior and middle frontal gyrus, and they are mainly located in the DLPFC and FPC regions. These brain regions show a particular relevance between the reward-sensitive personality trait and extraneous novel stimuli.

## Discussion

### Relationship Between Reward Sensitivity and Passion

To our knowledge, this is the first fNIRS study to explore the interaction between the reward-sensitive personality trait and passion as a component of love. Our results showed that the activation of the triangular part of the inferior frontal gyrus whose functional brain region is in the VLPFC was significantly greater in individuals with high reward sensitivity than in those with low reward sensitivity when looking at their partner’s photograph, suggesting that there are neurophysiological differences between reward sensitivity and passionate arousal. Moreover, individuals with high reward sensitivity showed greater activation of the triangular part of the inferior frontal gyrus when looking at their partners compared to strangers and friends. This indicated that the influence of familiarity on activation patterns could be excluded, verifying the specificity of the triangular part of the inferior frontal gyrus in passionate arousal relating to partners. The results of our study support the hypothesis that individuals with different reward sensitivities show differences in prefrontal cortex activation patterns under passionate arousal.

The lateral prefrontal cortex consists of the VLPFC, the DLPFC, and the orbitofrontal cortex. It is an important brain region that integrates emotional experience and the control of emotional information in goal-directed behavior (Gyurak et al., 2011; Ochsner et al., 2012; Gu et al., 2018). Individuals with high activation of the VLPFC perform better in the reappraisal of aversive images, and this activation is negatively correlated with amygdaloid nucleus activity and positively correlated with ventral striatum activity (Wager et al., 2008). This suggests that the VLPFC has a control function in suppressing negative emotions (Ochsner et al., 2012). In recent years, some studies have focused on the functional role of the VLPFC in dealing with positive emotions, suggesting that VLPFC activation can lead to better emotional regulation in interpersonal conflict. Hooker et al. (2014) revealed that individuals with social anhedonia (SA) are associated with VLPFC brain damage affecting the control of positive emotion using a community sample of people with high and low social anhedonia. Individuals with high SA had less VLPFC activity in response to positive facial expressions compared with low SA. This low activity was significantly associated with low expectations for happiness, suggesting that the VLPFC is related with the function of processing positive social cues. Yin et al. (2015) further explored the connection neuromechanism between the VLPFC and emotional processing regions. The results indicated that individuals with high SA showed decreased connectivity between the VLPFC and motor cortex, inferior parietal lobe, and posterior temporal lobe when watching positive social videos, while low SA showed increased connectivity, indicating that the VLPFC plays a regulatory role in social and emotional processing, especially in the upregulation of responses to positive social information. The regulatory role affecting other brain regions is amplifying the positive information in social interactions or inhibiting the interference of other irrelevant emotional information (Hooker et al., 2014; Yin et al., 2015). Therefore, we believe that the passionate arousal in the VLPFC induced by photographs of their partner in individuals with high reward sensitivity is related to the amplification and processing of positive social information in this region. Our study supports the previous finding that the VLPFC has the function of cognitive control in emotional processing, integrating emotional experience and goal-directed emotional information (Gyurak et al., 2011; Ochsner et al., 2012). Our results further demonstrates that this cognitive control not only acts on emotional information but also embodies the object selection unique to those who are currently in love.

### Relationship Between the Reward Sensitivity and Novel Stimuli

Our interaction results also showed that individuals with high reward sensitivity had significant activation in the DLPFC and FPC compared to those with low reward sensitivity when looking at photographs of strangers. This indicates that these regions are sensitive to extraneous novel stimuli. The results of Study 1 have also found a positive correlation between reward sensitivity and the number of previous relationships, which is consistent with the hypothesis of the RST that individuals with high reward sensitivity tend to have higher levels of approaching behavior; hence, it may be easier for such individuals to start a new intimate relationship.

A video game training fMRI study showed that changes in game interest after training may be responsible for a decrease in DLPFC activity, suggesting that extraneous novel stimuli exploration is closely related to the DLPFC (Gleich et al., 2017). A study of repetitive transcranial magnetic stimulation of the DLPFC in 47 individuals with depression indicated that non-responders showed higher levels of pleasure loss (including pessimism, loss of pleasure, and loss of interest in previously enjoyed activities) and lower connectivity to the classic reward circuit (including the ventral tegmental area, striatum, and ventromedial prefrontal cortex) compared with responders, and compared to individuals reporting anhedonia, depressed individuals with retained pleasure functioning were also found to be sensitive to DLPFC transcranial magnetic stimulation, which indicates that the region is sensitive to reward information (Downar et al., 2014). Studies by Daffner et al. (2000) showed that DLPFC-injured individuals displayed decreased exploratory behavior toward novel and unusual stimuli and that the attenuation response to novel and unusual stimuli stimuli was significantly correlated with degree of apathy. These findings suggest that the DLPFC plays a critical part in directing and sustaining attention to novel and unusual events, and the impairment of novelty seeking behavior may contribute to the characteristic apathy found in individuals with DLPFC injury. Therefore, the DLPFC is known to be sensitive to reward information and novel stimuli, supporting our results of significant DLPFC activation when individuals with high reward sensitivity viewed photographs of strangers. Our findings also support RST, which posits that individuals with high reward sensitivity (strong BAS) are more easily affected by appetitive stimuli (conditioned and unconditioned appetitive signals of reward), leading to an increase in exploratory behavior.

Our interaction results also showed that individuals with high reward sensitivity had significant activation in the FPC compared to those with low reward sensitivity when looking at photographs of strangers. Functional imaging studies have suggested that the FPC is involved in the experience of prosocial sentiments (Zahn et al., 2009; Moll et al., 2011, 2014). An fNIRS study found that the frontal and superior temporal regions were activated during face-to-face conversation, with higher activity in the speaking segments than in the mute segments during conversation, particularly in frontopolar NIRS channels (Suda et al., 2010). Individuals with FPC lesions were observed to behave inappropriately and less prosocially, which could be attributed to a generalized emotional blunting. Moll et al. (2011) investigated the brain regions distinctively associated with sentiment impairments in individuals with the behavioral variant of frontotemporal dementia, and the results showed that the degree of impairment of prosocial sentiments was associated with the degree of damage to the frontopolar cortex. Another fMRI study in healthy participants also suggested that people can voluntarily enhance their brain signatures of tenderness/affection, which are related to the function of promoting prosocial emotions in the FPC and septohypothalamic circuit (Moll et al., 2014). Combined with our results, the exploration of novel stimuli or curiosity toward strangers of highly reward-sensitive individuals might also be related to the prosocial function of the FPC.

In addition, comparison of the activity in three photo types showed greater activation mainly in the prefrontal cortex region when the subjects were viewing partner photographs compared with stranger photographs. The results revealed that the prefrontal cortex responds more strongly to familiar photos than to non-familiar photos, which is consistent with previous studies (Johnston and Edmonds, 2009; Taylor et al., 2009).

### Limitations

There are a few limitations to our study. First, this study only used college students as subjects, and thus this sample only represents part of the population. In future research, community sample of people could be considered to improve the representativeness of the samples in the research of intimate relationships. Second, the PLS questionnaire was not used in study 2. The combination of the self-report and neurophysiological measurements of passion would more effectively reveal the relationship of reward sensitivity and passion. Finally, researchers in both neuroimaging and behavioral research widely believe that passion is time-dependent, liable to diminish over time (Ahmetoglu et al., 2010; Miller, 2011). Future research using a longitudinal approach could evaluate and analyze the individual differences and changes in passion over time.

## Conclusion

Our results show that the individual reward-sensitive personality trait is only associated with passion, and the individual punishment-sensitive personality trait is associated with intimacy and commitment but not passion in intimate relationships. We found significant interactions between reward sensitivity and photograph type, and the triangular part of the inferior frontal gyrus showed a particular relevance to the reward-sensitive personality trait toward partners. These results also support RST, indicating that the reward-sensitive personality trait is associated with passion in intimate relationships. Exploration of the mechanisms underlying reinforcement-sensitive personality traits related to passion enhances our understanding of heterosexual intimate relationships. Furthermore, this work has important implications for the research methodology and theory of intimate relationships and may also contribute to clinical applications such as family therapy.

## Data Availability Statement

The datasets generated for this study are available on request to the corresponding author.

## Ethics Statement

The studies involving human participants were reviewed and approved by the Ethics Committee of Tianjin Normal University, and the pilot study was performed strictly in accordance with the approved guidelines. The patients/participants provided their written informed consent to participate in this study.

## Author Contributions

LG, RY, and XB designed the work. LG and RY wrote the manuscript. LG, RY, and PZ acquired the data. LG, RY, and QZ analyzed the data. All authors contributed to the article and approved the submitted version.

## Conflict of Interest

The authors declare that the research was conducted in the absence of any commercial or financial relationships that could be construed as a potential conflict of interest.
