# Supplementary material for: Reinforcement-Sensitive Personality Traits Associated With Passion in Heterosexual Intimate Relationships: An fNIRS Investigation
Source: Front Behav Neurosci. 2020 Jul 21;14:126. doi: 10.3389/fnbeh.2020.00126 (PMC7385243; doi:10.3389/fnbeh.2020.00126)
Supplement: Supplementary file 1 [file Table_1.docx]

**Supplementary Material**

Correspondence between fNIRS channel layout and Anatomical Automatic Labeling (AAL) brain regions

| Channel | MNI | | | AAL | Hemisphere | Maximum overlap |
| --- | --- | --- | --- | --- | --- | --- |
|  | X | Y | Z |  |  |  |
| 1 | 61 | 10 | 34 | Precentral | right | 66.67% |
| 2 | 48 | 37 | 34 | Frontal_Mid | right | 82.20% |
| 3 | 31 | 55 | 32 | Frontal_Mid | right | 76.55% |
| 4 | 14 | 64 | 33 | Frontal_Sup_Medial | right | 48.67% |
| 5 | −10 | 62 | 36 | Frontal_Sup_Medial | left | 52.45% |
| 6 | −28 | 55 | 32 | Frontal_Mid | left | 58.90% |
| 7 | −44 | 39 | 33 | Frontal_Mid | left | 91.10% |
| 8 | −56 | 13 | 34 | Precentral | left | 50.90% |
| 9 | 68 | 0 | 23 | Postcentral | right | 72.01% |
| 10 | 57 | 31 | 23 | Frontal_Inf_Tri | right | 93.08% |
| 11 | 44 | 53 | 24 | Frontal_Mid | right | 100.00% |
| 12 | 24 | 67 | 24 | Frontal_Sup | right | 71.05% |
| 13 | 4 | 67 | 25 | Frontal_Sup_Medial | right | 51.18% |
| 14 | −21 | 66 | 25 | Frontal_Sup | left | 97.71% |
| 15 | −40 | 54 | 23 | Frontal_Mid | left | 99.57% |
| 16 | −54 | 32 | 23 | Frontal_Inf_Tri | left | 93.99% |
| 17 | −65 | 1 | 25 | Postcentral | left | 70.61% |
| 18 | 62 | 20 | 12 | Frontal_Inf_Tri | right | 48.08% |
| 19 | 52 | 46 | 13 | Frontal_Mid | right | 61.29% |
| 20 | 36 | 64 | 14 | Frontal_Mid | right | 53.44% |
| 21 | 16 | 72 | 16 | Frontal_Sup | right | 49.81% |
| 22 | −13 | 72 | 15 | Frontal_Sup | left | 74.26% |
| 23 | −33 | 63 | 14 | Frontal_Mid | left | 60.53% |
| 24 | −49 | 47 | 13 | Frontal_Mid | left | 47.25% |
| 25 | −59 | 22 | 15 | Frontal_Inf_Tri | left | 70.51% |
| 26 | 68 | −3 | −9 | Temporal_Sup | right | 55.33% |
| 27 | 58 | 37 | 1 | Frontal_Inf_Tri | right | 66 .12% |
| 28 | 46 | 58 | 2 | Frontal_Mid | right | 57.54% |
| 29 | 27 | 70 | 4 | Frontal_Sup | right | 80.33% |
| 30 | 4 | 71 | 6 | Frontal_Sup_Medial | right | 50.32% |
| 31 | −22 | 71 | 6 | Frontal_Sup | left | 91.30% |
| 32 | −42 | 59 | 2 | Frontal_Mid | left | 57.94% |
| 33 | −55 | 37 | 2 | Frontal_Inf_Tri | left | 74.10% |
| 34 | −65 | −2 | −6 | Temporal_Sup | left | 53.74% |
| 35 | 61 | 10 | −19 | Temporal_Pole_Mid | right | 42.86% |
| 36 | 52 | 48 | −9 | Frontal_Inf_Orb | right | 64.23% |
| 37 | 38 | 65 | −7 | Frontal_Mid_Orb | right | 61.15% |
| 38 | 16 | 73 | −5 | Frontal_Sup_Orb | right | 45.81% |
| 39 | −13 | 73 | −4 | Frontal_Sup_Orb | left | 36.30% |
| 40 | −34 | 65 | −5 | Frontal_Mid_Orb | left | 59.19% |
| 41 | −50 | 48 | −7 | Frontal_Inf_Orb | left | 59.48% |
| 42 | −58 | 11 | −16 | Temporal_Pole_Sup | left | 50.71% |

MNI: Montreal Neurological Institute
